# Phylogenomics illuminates the phylogeny of flower weevils (Curculioninae) and reveals ten independent origins of brood-site pollination mutualism in true weevils

**DOI:** 10.1098/rspb.2023.0889

**Published:** 2023-10-11

**Authors:** J. Haran, X. Li, R. Allio, S. Shin, L. Benoit, R. G. Oberprieler, B. D. Farrell, S. D. J. Brown, R. A. B. Leschen, G. J. Kergoat, D. D. McKenna

**Affiliations:** ^1^ CBGP, CIRAD, INRAE, IRD, Institut Agro, Univ. Montpellier, Montpellier, France; ^2^ Department of Entomology, College of Plant Protection, China Agricultural University, Beijing 100193, People's Republic of China; ^3^ Department of Biological Sciences, University of Memphis, Memphis, TN 38152, USA; ^4^ Center for Biodiversity Research, University of Memphis, Memphis, TN 38152, USA; ^5^ CBGP, INRAE, IRD, CIRAD, Institut Agro, Univ. Montpellier, Montpellier, France; ^6^ School of Biological Sciences, Seoul National University, Seoul 08826, Republic of Korea; ^7^ CSIRO, Australian National Insect Collection, GPO Box 1700, Canberra, Australian Capital Territory 2601, Australia; ^8^ Department of Organismic and Evolutionary Biology, Harvard University, Cambridge, MA, USA; ^9^ Bio-Protection Research Centre, Lincoln University, P.O. Box 85084, Lincoln 7647, New Zealand; ^10^ Manaaki Whenua—Landcare Research, New Zealand

**Keywords:** brood-site pollination, convergent evolution, insect–plant interactions, systematics, weevils, exon capture

## Abstract

Weevils are an unusually species-rich group of phytophagous insects for which there is increasing evidence of frequent involvement in brood-site pollination. This study examines phylogenetic patterns in the emergence of brood-site pollination mutualism among one of the most speciose beetle groups, the flower weevils (subfamily Curculioninae). We analysed a novel phylogenomic dataset consisting of 214 nuclear loci for 202 weevil species, with a sampling that mainly includes flower weevils as well as representatives of all major lineages of true weevils (Curculionidae). Our phylogenomic analyses establish a uniquely comprehensive phylogenetic framework for Curculioninae and provide new insights into the relationships among lineages of true weevils. Based on this phylogeny, statistical reconstruction of ancestral character states revealed at least 10 independent origins of brood-site pollination in higher weevils through transitions from ancestral associations with reproductive structures in the larval stage. Broadly, our results illuminate the unexpected frequency with which true weevils—typically specialized phytophages and hence antagonists of plants—have evolved mutualistic interactions of ecological significance that are key to both weevil and plant evolutionary fitness and thus a component of their deeply intertwined macroevolutionary success.

## 1. Introduction

Flowering plants have evolved a diversity of strategies to attract pollinators. Among these strategies, brood-site (or nursery) pollination is a peculiar mutualism in which the plant provides a brood-site to the insect as a reward for being pollinated [1]. This intimate interaction begins early in the life cycle of the insect because the larva develops directly in the host plant tissue. Fig-wasps pollinating the flowers of *Ficus* (Moraceae) constitute a textbook example of brood-site pollination [2–5]. Brood-site pollination mutualisms are known to involve insects in the orders Coleoptera, Diptera, Hymenoptera, Lepidoptera and Thysanoptera and a diversity of plant lineages [1,6–8]. Such interactions are often highly specialized and provide opportunities to investigate the evolutionary dynamics of plant–insect interactions [8].

With more than 62 000 known species, weevils (Coleoptera: Curculionoidea) comprise the most species-rich radiation of phytophagous beetles [9]. Notably, they also exhibit an extraordinary diversity of brood-site pollination interactions with plants [10–12]—far more than any other group of insects. Approximately 250 plant species belonging to 72 genera (including a diversity of gymnosperms, eudicots and monocots) have been documented to exhibit brood-site pollination interactions with approximately 300 species of weevils. These ecologically important mutualisms are mainly documented from tropical and subtropical biomes [12]. The overall picture of this mutualistic system is far from being complete—and thus, our estimates of their number are certain to be substantial underestimates—because new brood-site pollination interactions are regularly reported in the scientific literature [13] and entire clades of weevils engaged in these interactions are still awaiting formal description (e.g. [10,11,14]. In our current knowledge, plant–weevil pollination mutualisms involving brood-sites have evolved independently at least 12 times across different weevil families [12]. These mutualisms sometimes involve comparatively ancient weevil and plant lineages, such as the weevil family Belidae in association with cycads in the family Zamiaceae; however, the actual taxa involved (weevil subtribe Allocorynina and cycad genera *Dioon* and *Zamia*) are much younger diversifications [15–17] and the temporal origins of their mutualisms are therefore also indicated to be relatively recent. In true weevils, the evolutionary dynamics of host preference inferred in a tribe of flower weevil pollinators (Derelomini) suggests that new associations among pollinator clades have also emerged fairly recently, during the Pliocene, approximately 5 million years ago (specifically, between *Derelomus* Schoenherr and Ebenaceae [18]).

Weevils involved in pollination mutualism often display a high level of host plant specificity at the species level and host plant consistency at the genus level [10]. However, host plant associations in Derelomini appear to be less constrained at deeper evolutionary scales than in other insect lineages involved in co-evolution or co-speciation, because shifts between unrelated plant lineages have been inferred [4,5,7,18]. Thus, weevils provide a potentially informative model for inferring the ecological and evolutionary processes underlying the evolution of brood-site pollination mutualism [19].

The vast majority of weevil lineages engaged in brood-site pollination are currently classified in the flower weevil subfamily Curculioninae, a globally distributed group of predominantly seed-feeders with about 4500 described species in 350 genera (table 1) [12,30–32]. However, the classification of the subfamily into natural groups is inconsistent and controversial, and none of the molecular phylogenetic studies of weevils conducted to date have produced a robust and consistent phylogenetic hypothesis nor used a dense enough sampling of Curculioninae to elucidate the relationships among the flower weevils [15,33–37]. Curculioninae in the current concept [32] are frequently recovered as a polyphyletic group with high statistical support [15,35–38], their relationships with other curculionid subfamilies in the CCCMS clade (Conoderinae, Cossoninae, Curculioninae, Molytinae and Scolytinae [37]) are not yet resolved and no morphological characters have been identified as synapomorphies for the Curculioninae. Thus, the current concept of Curculioninae (and of the tribes included in it) is based largely on vague morphological data, the interpretation of which varies, sometimes considerably so, among authors. Many weevil groups traditionally comprising Curculioninae lack relevant apomorphic features [32]. For example, 18 of the 34 tribes of Curculioninae included in a recent comprehensive revision lack clear boundaries, three are monotypic and could not be related to any existing groups of Curculioninae and 12 genera were treated as *incertae sedis* [32]. This ambiguity applies to several tribes that have brood-site pollinators. For instance, despite Derelomini being the most well-studied group of flower weevils, the relationship between Eastern and Western Hemisphere genera placed in the tribe remained unclear [14,32,39].

**Table 1.** Classification and hosts of the weevil genera included in this study that are known to engage in plant brood-site pollination mutualism. Genera marked with * contain species formally involved in brood-site pollination (experimental demonstration), others are suggested based on ecology, behaviour, host plant morphology and phenology; see details in Haran *et al*. [12]. For the genera marked with **, the specific species engaged in brood-site pollination was included in the sampling. For the genera marked with ^†^, only representatives of the same tribe or subfamily were included. *Systenotelus* is indicated as a representative of Derelomini, but this genus is not involved in brood-site pollination of its host.

higher rank	genera	hosts	key references
Curculioninae			
Derelomini	Derelomus** Schoenherr	Arecaceae; Ebenaceae; Fabaceae; etc	Düfay & Anstett [6]; Anstett [20]
	Cotithene* Voss	Cyclanthaceae	Valente *et al.* [21]
	Grasidius** Champion	Arecaceae	Auffray *et al.* [22]
	*Ebenacobius* Haran	Ebenaceae	Haran *et al.* [18,23]
	Elaeidobius** Kuschel	Arecaceae	Syed [24]
	*Notolomus* LeConte	Arecaceae	Brown [25]
	Perelleschus** Wibmer & O'Brien	Cyclanthaceae	Franz & O'Brien [26]
	*Systenotelus* Anderson & Gomez	Cyclanthaceae	Franz & Valente [10]
Eugnomini	Udeus*^†^ Champion	Urticaceae	Mendonça [27]
Ochyromerini	Endaeus* Schoenherr	Annonaceae	Dao *et al.* [28]
Storeini	Elleschodes* Blackburn	Eupomatiaceae	Amstrong & Irvine [29]
Molytinae			
Molytini	Tranes** Schoenherr	Zamiaceae	Toon *et al.* [11]
Amophocerini	Porthetes** Schoenherr	Zamiaceae	Toon *et al.* [11]
Baridinae	Montella*^†^ Bondar	Orchidaceae	Nunes *et al.* [13]

Therefore, the evolutionary history of true weevils remains poorly understood, and hypotheses underlying the emergence of specific lifestyles have not yet been addressed. In the case of weevil pollinators, it is unclear (i) whether the brood-site pollination lifestyle emerged early in the diversification of true weevils and persisted in only a small number of clades of Curculioninae, and if so, (ii) whether this pollination mechanism also re-emerged relatively recently (secondarily) in Curculionidae and in many groups independently [14,40,41]. Under the first scenario, plant-weevil pollination mutualism would be primarily an ancient reproductive system, as suggested by the fact that the involved plant families are predominantly ancient tropical lineages [12,42]. By contrast, the latter scenario would reflect a more dynamic system wherein weevils engage in mutualistic relationships at more recent evolutionary scales [18,43]. Also unknown in both situations are the lifestyle strategies that promoted the emergence of this ecologically and evolutionarily significant mutualism. The larvae of Curculioninae develop mostly in the reproductive tissues of their host plants [9,32], a condition that is thought to have promoted the shift from parasitism to mutualism. The evolution of mutualism, in this case, relates to the behaviour of adults, which move between inflorescences of conspecific plant species to mate and oviposit, potentially pollinating them [12]. This assertion, however, requires confirmation because some cycad-associated weevils (Amorphocerini) have been postulated to have switched from an ancestral lifestyle of trunk-boring to brood-site pollination mutualism, suggesting that alternative pathways to pollination mutualism have evolved [41].

To test these hypotheses, we reconstructed the phylogenetic relationships and evolutionary dynamics of host use in weevil tribes involved in brood-site mutualism in the context of a dense, global sampling of weevils belonging to the flower weevil subfamily. We first evaluated the monophyly of the currently recognized tribes and assessed their interrelationships in Curculioninae to determine the level of phylogenetic conservatism associated with brood-site mutualism. Then, to gain insights into the transitions associated with the emergence of brood-site pollination mutualism, we inferred the ancestral lifestyle character states for the larvae of the weevils included in this study.

## 2. Material and methods

### (a) Taxon sampling

Twenty-nine tribes of Curculioninae *sensu* Caldara *et al*. [32] were sampled, representing approximately 85% of the 34 currently recognized, with 1–20 genera sampled from each tribe, including the type genera in 27 cases. Six additional genera currently classified as *incertae sedis* in Curculioninae were also included. Representatives of other subfamilies in the CCCMS clade (*sensu* [44] and [37], all but Conoderinae, including type genera) were also included, as previous phylogenetic reconstructions reported unresolved relationships between them and Curculioninae [15,33,35–37,45]; furthermore, several lineages of brood-site pollinators are known from these subfamilies (table 1; see also [12] for a review). The sampled weevil species included representatives of most weevil lineages engaged in brood-site pollination [32]; table 1). We also included a few genera for which this behaviour is assumed but not verified to date (e.g. *Acalyptus* Schoenherr, *Eudelodes* Zimmerman, *Notolomus* LeConte) and genera of lineages containing brood-site pollinators but whose species actually pollinating their hosts could not be obtained for this study (*Endaeus* Schoenherr, *Elleschodes* Blackburn, *Udeus* Champion). In all, the ingroup selection included a worldwide sample of six weevil subfamilies, 44 tribes and 130 genera, including 14 genera (10%) with species known to engage in plant brood-site pollination mutualism (table 1, see specimen details in electronic supplementary material, table S1).

Selection of outgroups was based on previously inferred relationships for weevils [15,35,37]. The following closely related outgroups from the CEGH clade (Cyclominae, Entiminae, Gonipterini, Hyperinae) (‘broad-nosed’ weevils; see [37] were sampled: Cyclominae (four tribes, seven genera), Entiminae (11 tribes, 12 genera) and Hyperinae (two tribes, three genera). More distant outgroups were chosen from the curculionid subfamilies Brachycerinae and Dryophthorinae and the family Brentidae. Vouchers of specimens newly sequenced were mounted, dried and deposited in the Continental Arthropod Collection at Centre de Biologie pour la Gestion des Populations, Montpellier, France (CBGP doi:10.15454/D6XAKL) or are maintained as part of the 1 K Weevils Project voucher collection in the McKenna Lab at the University of Memphis (Memphis, TN, USA).

### (b) DNA extraction, library preparation and sequencing

Evolutionary relationships were inferred in a phylogenomic framework using anchored hybrid enrichment (AHE) probes developed for Coleoptera [15,46], especially Phytophaga [15,47]. This set of probes targets 522 highly conserved nuclear protein-coding genes with more variable flanking regions. Data were newly generated for 146 of the 202 specimens/species included in this study. Data for the other 56 specimens/species have been previously published [15,47,48]. Our DNA extraction, library preparation and sequencing protocols followed Shin *et al*. [15]. Briefly, tissues from 96 ethanol-preserved or dry collection specimens (see Supplementary Information 2) were extracted non-destructively using an EZ-10 96-well plate DNA Kit (Biobasic Inc., Canada) with an overnight lysis step. Genomic DNA was sonicated at equal quantities to fragment sizes of 300–600 bp on a Bioruptor Pico (Diagenode; 15 s ON, 90 s OFF, eight cycles). Library enrichment was performed following the user manual of the NEBNext Ultra II DNA Library Prep Kit for Illumina (New England Biolabs, Ipswich, MA, USA). Each library was inline indexed and we applied 15 PCR cycles for the final enrichment. We pooled 16 libraries per pool at equal quantities and used myBaits Hyb Capture kits for AHE, following manufacturer instructions (Arbor Biosciences, Ann Arbor, MI, USA). The AHE libraries were paired-end (PE) sequenced on a 2 × 150 bp SP lane using an Illumina NovaSeq sequencer (Illumina, San Diego, CA, USA) at Montpellier GenomiX platform (MGX).

### (c) Assembly, extraction and analysis of phylogenetic markers

For all samples included in this study, read cleaning, assembly, orthologue prediction and cross-contamination cleaning followed the pipeline of Breinholt *et al*. [49]. In brief, this pipeline uses a probe-baited iterative assembly that extends beyond the probe region, checks for quality and cross-contamination due to barcode leakage, removes paralogues and returns a set of aligned orthologues for each locus and taxon of interest. Raw reads were assembled using an iterative baited assembly (IBA) after filtering with Trim Galore! v.0.4.0 (bioinformatics.babraham.ac.uk). Orthology was determined using the *Tribolium castaneum* genome (GCA_000002335.3) as a reference, and single-hit and genome mapping location criteria were used with NCBI Blastn [50]. Cross-contamination checks were conducted with USEARCH [51], and sequences with >99% identity across different subfamilies were identified and removed. Cleaned sequences were aligned in MAFFT v.7.245 [52], and isoform consensuses were generated using FASconCAT-G 1.02 [53]. Two datasets were created as follows: a first dataset composed of alignments including only the probe regions of each targeted gene, hereafter called probe-based alignments (PBA), and a second dataset including full-length alignments (i.e. probe region + flanking regions of each targeted gene), hereafter called full-length alignments (FLA).

Following Li *et al*. [54], a long-branch detection protocol was used to investigate the possibility of external contamination, paralogous sequences and/or large sequencing/assembly errors (*longbranchpruner.pl* available on Osiris, http://galaxy-dev.cnsi.ucsb.edu/osiris/). First, AliView v1.18 [55] was used to manually check each nucleotide probe-based alignment to ensure the probe region is in the correct open reading frame (ORF). Then, based on the nucleotide (NT) multiple sequence alignment of the probe-based alignments, gene trees (for each probe) were inferred using IQ-TREE v.2.1.3 [56], conducting a full model test for each probe region. Tip sequences that exceeded eight standard deviations from the mean tip length (species tree) of the gene tree were pruned from the full-length alignments. Additionally, to check for possible contamination in old samples (e.g. museum specimens), we also screened for traces of multiple mitochondrial genomes using MitoFinder V.1.4.1 [57]. Each mitochondrial fragment recovered was identified using BLAST on the GenBank database [58] and a laboratory-hosted database (source and result available from Zenodo). Two samples containing mitochondrial fragments clustering with distinct species were discarded (*Ita chavanoni* Meregalli & Borovec, Itini, JHAR03289; *Anchonocranus oleae* Marshall, *incertae sedis*, JHAR02096; both are not included in the species count above).

### (d) Supermatrix construction and inferences

Cleaning of non-homologous sequences and dubious parts of full-length alignments was performed with HMMcleaner using a threshold value of 9. At this stage, marker sequences included both probe sequences (known to be coding sequences) and flanking regions, but the alignment was lost due to the HMMcleaner procedure. To be able to treat the probe region and the flanking regions differently, each marker was then realigned with its associated probe sequence using MUSCLE. Flanking regions that could not be aligned with confidence were excluded from the matrix. Nucleotide sites presenting more than 50% of gaps were removed using a custom Perl script (source and result available from Zenodo). Loci found in less than 70% of species were excluded from further analyses. Based on the alignment with the probe sequence, up to three partitions were created for each full-length alignment, corresponding to the 3′ flanking region, the probe region and the 5′ flanking region. Partitions with less than 20 nucleotides were discarded from further analyses. This partitioning allowed us to perform different alignment strategies for probe and flanking regions. While flanking regions were aligned with MAFFT (using default settings [52], probe regions were aligned using the pipeline implemented in OMM_MACSE [59], version 11.05b). The latter is specifically developed for aligning coding sequences and ensuring the open reading frame is respected. Then, for each marker, flanking and probe regions were concatenated and gene tree inferences were performed with IQ-TREE based on up to five partitions, corresponding to one partition per flanking region and three codon partitions for the coding probe region. For each full-length alignment, a heuristic search was performed in IQ-TREE with a small perturbation strength (*-pers 0.2*) and ModelFinder (option *-m MFP + MERGE*); this allowed us to determine further the best partition scheme based on the Bayesian information criterion (BIC). We identified and applied the best-fitting models to the following phylogenetic reconstructions performed in IQ-TREE with 100 separate heuristic searches. We applied nearest-neighbor interchange (NNI) branch swapping to improve the tree search and limit overestimating statistical measures of nodal support due to severe model violations (‘-*bnni*’ command). Nodal support was computed using 1000 ultra-fast bootstrap (‘*-B*’ command) replicates [60,61] and SH-like approximate likelihood ratio tests (SH-aLRT; ‘*-alrt*’ command) [62]. Nodes with ultra-fast bootstrap values (uBV) higher than 95% and/or SH-aLRT values higher than 80% were considered robust. Dating analyses were not undertaken on this dataset due to a lack of well-characterized fossils for calibration in the CCCMS clade [63] and due to the generally poor performance of outgroup calibrations on ingroup ages (making this approach unsatisfying) in analyses of a previous AHE dataset for weevils [15].

### (e) Ancestral character state reconstruction

We used ancestral character state estimation (ASE) to reconstruct the use of specific plant tissues by larvae of weevils and to help illuminate the conditions that promoted the emergence of brood-site pollination in the CCCMS clade. Brood-site pollination was characterized and scored at the genus level (with either ‘yes’ or ‘no’) based on the results of a recent review [12], where brood-site pollination is either inferred based on ecology, behaviour, host plant morphology and phenology or based on formal experimental demonstrations (table 1). For the paraphyletic genus *Endaeus*, only one instance of brood-site pollination was coded in the corresponding ASE analysis to limit the risk of overestimating the number of independent origins of brood-site mutualism. Tissue specialization of larvae was categorized into the following lifestyles: (i) development in reproductive structures (seeds, fruits, ovaries, flower structures, pollen), (ii) leaf mining, (iii) development on leaves, (iv) development in root systems, (v) development in stems and (vi) development in dead wood.

As a guide tree, we used the best ML tree inferred with IQ-TREE; this tree was further modified in Mesquite v3.70 [64] by removing all taxa except those belonging to the CCCMS clade. For each character, species for which states could not be unequivocally determined were removed from the dataset by pruning the corresponding terminal branches in the guide tree; following this treatment, 141 terminals were retained for the analyses of brood-site pollination and 129 terminals were retained for the analyses of tissue specialization of larvae. Ancestral character state estimation (ASE) was further carried out with the *phytools* [65] package in R [66] by fitting and comparing different rate transition matrices of the Markov *k* state (*Mk*) model for discrete characters. The performances of three *Mk* models were compared: (i) the equal-rates model (ER), where a single parameter governs all transition rates; (ii) a symmetric model (SYM), where forward and reverse transitions share the same parameter; and (iii) an all-rates-different model (ARD), where each rate is a unique parameter. For the evolution of brood-site pollination, as it is a binary trait, the ER model is equivalent to the SYM model, so only two models (ER and ARD) were compared. All models were further fitted on the pruned tree with the *fitMk* function, and the best-fit model was selected based on AIC weights (*aic.w* function). Finally, ASE analyses with the best-fit model were conducted using a continuous-time-reversible Markov model with the *make.simmap* function with 1000 simulations.

## 3. Results

### (a) Phylogenetic analyses

**Figure 1.**
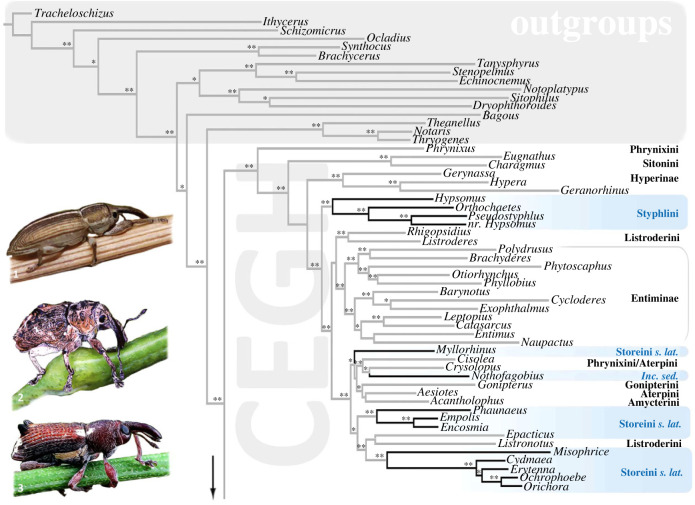
Maximum-likelihood tree resulting from analyses of 214 nuclear protein-coding genes (focus on the CEGH clade and outgroups). Support at node refers to SH-aLRT values ≥ 80% and uBV ≥ 95% (**). A single asterisk * refers to SH-aLRT values ≥ 80% only. Clades with black branches and highlighted in blue are classified in Curculioninae *sensu* Caldara *et al*., [32]. Taxa displayed on the left: 1 *Hypsomus* sp. (Styphlini); 2 *Myllorhinus* sp. (Storeini *s. lat.*); 3 *Encosmia* sp. (Storeini *s. lat.*).

**Figure 2.**
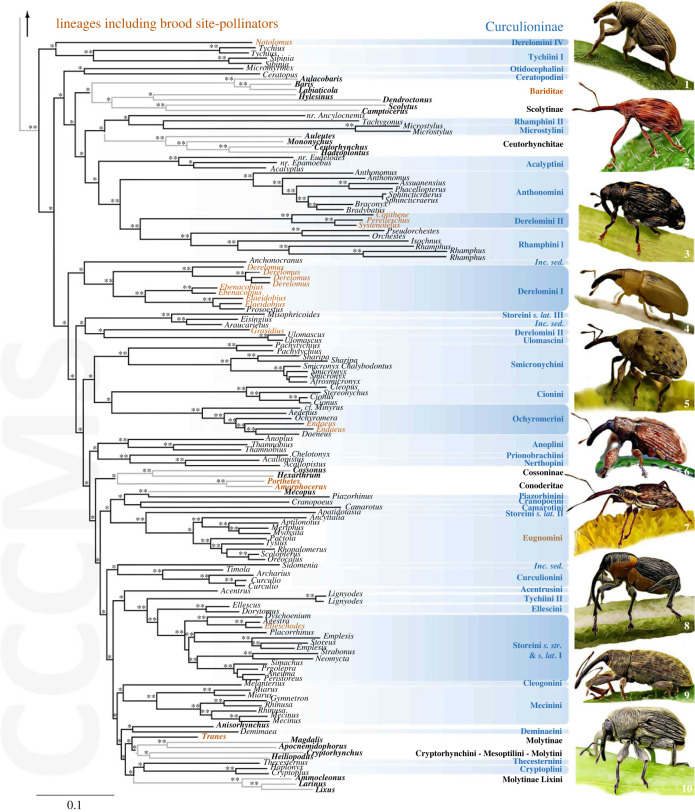
Maximum-likelihood tree resulting from analyses of 214 nuclear protein-coding genes (focus on the CCCMS clade). Node support values refer to SH-aLRT values ≥ 80% and uBV ≥ 95% (**). Single * refer to SH-aLRT values ≥ 80% only. Clades with black branches and highlighted in blue are classified in Curculioninae *sensu* Caldara *et al*., [32]. Clades highlighted in darker blue contain genera engaged in brood-site pollination mutualism and the corresponding genera are highlighted in orange (higher taxonomic rank when specific genera are not included in the tree). Other lineages of the CCCMS clade are in bold font. Taxa displayed on the right: 1 *Tychius* sp. (Tychiini); 2 *Anthonomus* sp. (Athonomini); 3 *Tachyerges* sp. (Rhamphini); 4 *Derelomus* sp. (Derelomini); 5 *Cionus* sp. (Cionini); 6 *Daeneus* sp. (Ochyromerini); 7 *Meriphus* sp. (Eugnomini); 8 *Archarius* sp. (Curculionini); 9 *Dorytomus* sp. (Ellescini); 10 *Cleopomiarus* sp. (Mecinini).

In total, 214 loci were retained in the alignment (56 509 bp, approx*.* 22% of missing data), corresponding to *ca.* 29 900 variable sites and 26 500 parsimony-informative sites. Overall branch support for ML analyses was high for SH-aLRT (94.5% of nodes ≥ 80% and 78.4% = 100%) and moderate for uBV (66.3% of nodes ≥ 95% and 58.3% = 100%; figures 1 and 2; electronic supplementary material, S1 and S2). The topology inferred for deeper relationships among Curculionoidea was consistent with previous reconstructions [15,37,47], with the early-diverging lineages (Brentidae, Bagoinae, Brachycerinae, Dryophthorinae, Errirhininae, Platypodinae) recovered as successive sister lineages of a well-supported clade encompassing the more derived CEGH and CCCMS clades (SH-aLRT and uBV of 100%). These latter clades were both highly supported (SH-aLRT and uBV of 100%) and included representatives of the subfamilies traditionally recognized in them (Cyclominae, Entiminae, Gonipterini, Hyperinae / Curculioninae, Conoderinae, Cossoninae, Molytinae, Scolytinae, respectively) except for the Styphlini and some Storeini *sensu lato*. Formal transfers are not undertaken here due to limited sampling in the CEGH clade, but suggestions of classificatory changes based on the topologies inferred are attached in electronic supplementary material, S1.

The subfamily Curculioninae was recovered as a polyphyletic lineage intermixed with the other subfamilies in the CCCMS clade (figure 2). Node support in this clade was high for SH-aLRT, but the deeper nodes showed weaker support for uBV. The topology inferred revealed three main subclades in the CCCMS clade: (i) a first small clade (I; SH-aLRT of 100%, uBV of 73%) encompassing only a part of Tychiini (*Sibinia* Germar and *Tychius* Germar) and the genus *Notolomus* (Derelomini) and forming the sister group of the rest of the CCCMS clade; (ii) a second clade (II: SH-aLRT of 99.4%, uBV of 47%) encompassing several tribes of Curculioninae (mainly Acalyptini, Anthonomini, Derelomini (part), Microstylini and Rhamphini), part of Conoderinae (supertribes Ceutorhynchitae and Bariditae) and Scolytinae; (iii) a third large clade (III: SH-aLRT of 100%, uBV of 58%) including the remaining tribes of Curculioninae, part of Conoderinae (supertribe Conoderitae), Cossoninae and Molytinae. The tribes Anthonomini, Cionini, Curculionini, Eugnomini, Mecinini, Ochyromerini and Smicronychini classified in Curculioninae were monophyletic as sampled, with high support, but Derelomini, Rhamphini, Storeini and Tychiini were not monophyletic for the genera sampled. Regarding the tribes containing pollinators, the Derelomini were found to be polyphyletic, comprising four unrelated lineages corresponding to the subtribes Notolomina, Phyllotrogina and Derelomina of Franz [14] and *Grasidius* Champion placed separately from the rest of the genera. These results confirm the status of Acalyptini as a distinct tribe [32,39] and indicate that Derelomini *sensu stricto* are restricted to the Old World and that the New World Phyllotrogina constitute a different, separate lineage (see electronic supplementary material, S1 for further discussion). *Tranes* Schoenherr was found nested in a clade containing several tribes of Molytinae (Cryptorhynchini, Mesoptiliini and Molytini; SH-aLRT of 100%, uBV of 77%) and Amorphocerini (*Amorphocerus* Schoenherr and *Porthetes* Schoenherr) clustered with Cossoninae (SH-aLRT of 100%, uBV of 88%).

### (b) Ancestral character state estimation

The evolution of brood-site pollination mutualism was inferred at least eight times in the CCCMS clade through the corresponding ASE analyses (best-fit model: ER; AIC weight of 0.631). For the clades of pollinators with denser taxon sampling, this condition was recovered as the ancestral state in Derelomina (excluding *Grasidius*) and Phyllotrogina (*sensu* [14] but not in Ochyromerini. Ancestral states for the deepest nodes in the CCCMS clade were all inferred as non-pollinators with maximal support (electronic supplementary material, figure S3).

**Figure 3.**
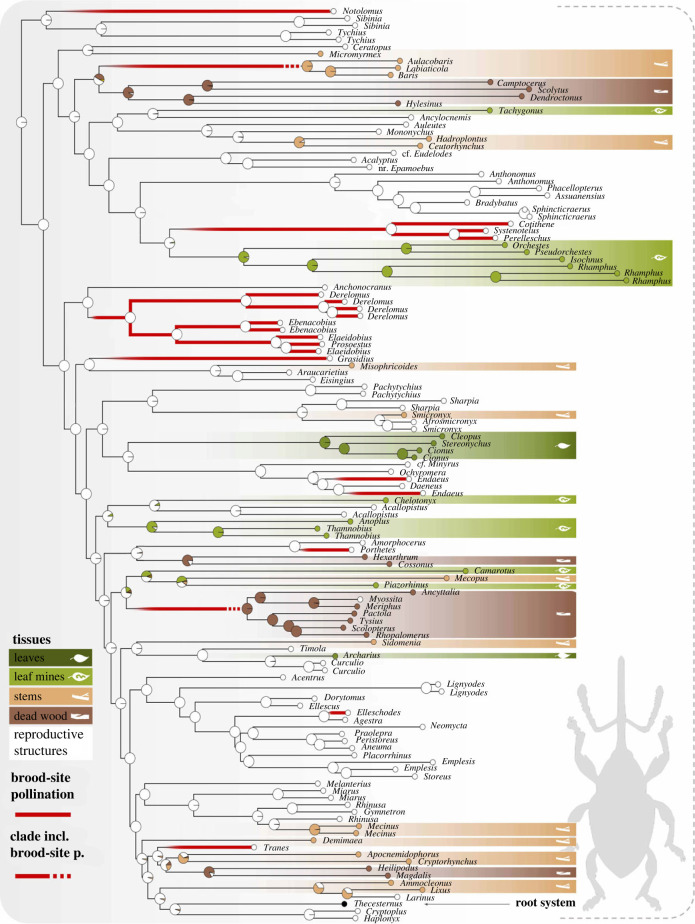
Results of the ASE analysis of larval tissue specialization carried out on the CCCMS clade, with an ER model and using a continuous-time reversible Markov model with 1000 simulations. In addition, red branches are used to underline the independent origins of brood-site mutualism inferred in another ASE analysis (see electronic supplementary material, figure S3). Two clades including brood-site pollinator genera that were not sampled in our study are also highlighted using red dotted lines. See figure 2 for classification of lineages.

The condition of larval development in the reproductive tissues of host plants was recovered as the ancestral state for the CCCMS clade as sampled (best-fit model: ER; AIC weight of 0.7254 versus 0.2745 for SYM and 2.4 × 10^−5^ for ER). From this condition, the ASE analysis inferred four transitions to development in dead wood, one to development on leaves, one to development in root systems, eleven to development in stems and five to leaf mining (figure 3; electronic supplementary material, figure S3). Larval specialization on reproductive structures was inferred as the ancestral state for all lineages with species engaged in brood-site pollination mutualism.

## 4. Discussion

### (a) Dismantling the concept of Curculioninae

This study constitutes the first formal molecular investigation of the phylogenetic relationships among the flower weevil subfamily. Based on a dense and worldwide sampling of lineages in this clade, we infer that the current concept of Curculioninae (*sensu* [32]) is not satisfactory, as it is unambiguously polyphyletic. Indeed, several lineages previously assigned to Curculioninae due to their long rostrum and endophytic larvae (typical conditions for CCCMS and Curculioninae in particular [9,32]) clustered in the CEGH clade, whose members generally have a short rostrum and ectophytic larvae [67] (see electronic supplementary material, S1). The condition of the elongated rostrum was previously reported in other genera of this clade, such as *Phrynixus, Gerynassa* and other Hyperinae and several genera of different tribes of Cyclominae [68]. Furthermore, many of the Australian genera here found to belong in the CEGH clade (*Cydmaea* Pascoe*, Empolis* Blackburn*, Encosmia* Blackburn*, Epacticus* Blackburn*, Erytenna* Pascoe*, Misophrice* Pascoe*, Ochrophoebe* Pascoe) and also the South African *Hypsomus* Schoenherr possess iridescent scales, probably due to three-dimensional photonic scales, which appear to be an autapomorphic character of the CEGH clade [69]. The inclusion in CEGH of all these genera with an elongated rostrum and endophytic larvae and their distribution in the phylogenetic tree suggest that these traits may also be plesiomorphic conditions in this clade and that the short rostrum and ectophytic larva of most Hyperinae, Entiminae and Cyclominae have evolved secondarily and several times. As with bark and ambrosia beetles [35,70], this provides an example of how the interpretation of similar but apparently convergent features (long rostrum and endophytic larva) has led to classifications of Curculioninae not reflecting phylogenetic relationships. A more definitive resolution of directionality in the evolution of these traits may be achieved in future studies by mapping these features on a more comprehensively sampled phylogenetic tree of the CEGH clade.

The topology inferred for the CCCMS clade also suggests that the generally well-supported tribes of Curculioninae are intermixed with other subfamilies (Conoderinae, Cossoninae, Molytinae and Scolytinae). Though the deeper nodes of the CCCMS clade were not fully resolved, it is clear that the concept of the flower weevil subfamily (Curculioninae) in its narrow or widest sense [30–32,71] requires substantial revision. This conclusion agrees with previous preliminary investigations of these relationships based on molecular data [15,33,35–37] and also emphasizes the challenge of identifying this subfamily based on adult and/or larval morphological characters [32,72]. A formal rearrangement of major clades is not undertaken here and postponed until the hard polytomy at the base of the CCCMS clade can be more definitively resolved and taxon sampling reflects the relative importance of all subfamilies clustering in this clade.

### (b) Multiple independent origins of brood-site pollination in weevils

All lineages that contain brood-site pollinators and were included in this study clustered into the CCCMS clade, which is consistent with the current classification of their subfamilies, as sampled in previous molecular phylogenies [15,32,37]. However, we did not recover a single lineage associated with this lifestyle. Instead, brood-site pollinators clustered into eight distinct clades of Curculioninae, and Derelomini alone constituted four distinct lineages, with Acalyptini confirmed as a further separate lineage [39]. When accounting for all lineages included in this study, ten events of the emergence of brood-site pollination mutualism are inferred, and this number reaches 15 when including weevil lineages not sampled here (Trypetidini; undet. Storeini, Curculioninae) and those outside the CCCMS clade (Belidae: Allocorynina) Brentidae, with only one species of *Antliarhinus* with a minor role in pollination [12]. Consequently, the number of independent origins of brood-site pollination mutualism is larger than previously thought [18,23]. The evolutionary dynamic of brood-site mutualism in weevils, therefore, sharply contrasts with those of other insect systems showing brood-site pollination, in which the current diversity appears to originate from only one colonization event of a host lineage by a specific insect family [4,5,8] (but see [7,73]). Our results reinforce the patterns observed in Derelomini, where the colonization of palms led to diversification in association with this plant lineage, followed by secondary shifts onto unrelated dicotyledonous lineages, sometimes including parallel colonizations [18].

The evolutionary and ecological context promoting repeated and extreme host shifts, such as those inferred in the case of brood-site pollination mutualisms involving weevil and plant life histories, is unclear, in particular in insects with endophytic larvae for which development is expected to be associated with host-specific physiological adaptations. In other brood-site pollination systems involving endophytic pollinators, host shifts have only been inferred within one plant family, which is expected to present more structural and physiological similarities in the brood-sites colonized [74–76]. Weevils engaged in brood-site interactions thus simultaneously exhibit highly specialized relationships with plants at the plant species level and on ecological timescales [12] and a remarkable ability to colonize new plant lineages on evolutionary timescales.

### (c) Transitions from detrimental to mutualistic relationships

The condition of larvae antagonistically associated with the reproductive structures of plants was recovered as the ancestral state in the CCCMS clade as sampled in this study. Reproductive plant organs (buds, flowers, fruits and seeds) are generally nutrient-rich substrates (although sometimes strongly defended by chemical compounds) that were possibly easier to use by larvae of early diverging weevil lineages in this clade. The other tissues used (stems, dead wood, leaves) are usually associated with specific adaptations or mutualism for larval development (e.g. galling, symbiosis with microorganisms, horizontal gene transfer conveying novel metabolic capabilities [48,77,78]. It should be noted, however, that this pattern may be biased due to the unbalanced sampling in favour of Curculioninae, whose lineages are predominantly associated with these tissues [32]. Because ancestral character state estimation analyses are sensitive to sampling bias, a definitive conclusion on the ancestral substrate of the CCCMS is pending a future assessment including more balanced sampling among the subfamilies in this clade.

The evolution of brood-site pollination in weevils was generally inferred as transitions from mostly detrimental associations with reproductive structures of plants to mutualism, a trend widely documented in brood-site pollination mutualistic systems [8,79]. In the case of weevils, the diversity of lineages developing in reproductive structures [9,32], and associated flower-visiting behaviour, was recently suggested as a context promoting the emergence of mutualism [12]. Indeed, adults in these lineages generally visit flowers to feed on pollen, mate and oviposit in buds, ovaries and fruits, in which larval development occurs. As these specialized beetles fly between host plant conspecifics to find new resources, they can carry pollen and potentially pollinate them. In specific environments such as tropical biomes, in which pollen limitation is a major constraint due to the absence or limited availability of non-specific pollination systems (anemophily and generalist entomophily [80–82]), such behaviour probably facilitated the emergence of specialized pollination systems such as brood-site pollination mutualisms.

## 5. Conclusion

This study provides a first assessment of the phylogenetic relationships of the subfamily Curculioninae and highlights the need for a complete recasting of the classification of the ‘true weevils’. The topology inferred further reveals the unique ability of higher weevils to engage in novel pollination mutualisms with plants. This pattern originates predominantly in their ancestral association with the reproductive structures of plants and a remarkable propensity to shift between host plant lineages.

## Data Availability

Illumina reads have been submitted to the Short Read Archive (SRA) of the National Center for Biotechnology Information (NCBI) and are available under BioProject number PRJNA1021960. AHE assemblies, phylogenetic dataset, corresponding tree and other supplementary materials are available from Zenodo [83]. The data are provided in electronic supplementary material [84].
